# Valorization of Olive Oil and Wine Industry Byproducts: Challenges and Opportunities in Sustainable Food Applications

**DOI:** 10.3390/foods14142475

**Published:** 2025-07-15

**Authors:** María Rodríguez-Pérez, Beatriz García-Béjar, Emma Burgos-Ramos, Paula Silva

**Affiliations:** 1 Biochemistry Area, Faculty of Environmental Sciences and Biochemistry, University of Castilla-La Mancha, Avenue Carlos III s/n, 45071 Toledo, Spain; maria.rodriguezperez@uclm.es (M.R.-P.); emma.burgos@uclm.es (E.B.-R.); 2 Department of Analytical Chemistry and Food Technology, University of Castilla-La Mancha, Avenue Carlos III s/n, 45071 Toledo, Spain; beatriz.gbermejo@uclm.es; 3 Laboratory of Histology and Embryology, Department of Microscopy, School of Medicine and Biomedical Sciences (ICBAS), University of Porto (U.Porto), Rua Jorge Viterbo Ferreira 228, 4050-313 Porto, Portugal; 4 iNOVA Media Lab, ICNOVA-NOVA Institute of Communication, NOVA School of Social Sciences and Humanities, Universidade NOVA de Lisboa, 1069-061 Lisbon, Portugal

**Keywords:** olive oil, wine, organic waste, bioactive compounds, functional foods, circular economy

## Abstract

The historical co-production of olive oil and wine has influenced the Mediterranean landscape and economy. Olive oil and wine production generates substantial organic waste, including olive pomace, grape pomace, and wastewater, which poses environmental challenges if untreated. These byproducts contain bioactive compounds, including polyphenols, such as hydroxytyrosol, resveratrol, and flavonoids, which possess antioxidant and anti-inflammatory properties, making them valuable for the development of functional foods and nutraceuticals. A combined waste valorization strategy can enhance bioactive compound recovery and align it with circular economic principles. The incorporation of olive oil and wine byproducts into food matrices, such as bread, pasta, dairy products, baked goods, chocolates, beverages, and processed items, has been explored to enhance antioxidant content, dietary fiber, and nutritional value. However, successful integration depends on maintaining acceptable sensory qualities and addressing the technical challenges in extraction, processing, and regulatory compliance. Realizing the potential benefits of dual valorization requires a systemic shift integrating scientific innovation, regulatory adaptability, and consumer engagement, guided by evidence, transparent communication, and inclusive governance to ensure that sustainability goals translate into environmental, economic, and public health outcomes.

## 1. Introduction

The historical co-production of olive oil and wine has significantly influenced the Mediterranean landscape and economy, establishing a distinctive agro-technical system grounded in dry farming practices and supported by favorable climatic conditions [1,2]. Grapevines and olive trees, alongside wheat, have long formed the agricultural triad of the region, providing not only dietary basics, but also goods of substantial symbolic, economic, and cultural value. Archaeological investigations have uncovered pressing installations where both oil and wine are processed, either sequentially or seasonally, demonstrating the flexibility of infrastructure and the complementary nature of these crops [3,4]. At sites such as Qlaʿ, production was organized on a large scale and designed for surplus, indicating a transition from subsistence farming to market-oriented agriculture, where the concurrent processing of grapes and olives facilitated optimized labor use and spatial efficiency [5]. These findings challenge the perception that wine and oil production are always segregated and support a model of integration that enhances resilience and technological continuity. This historical synergy is increasingly pertinent in contemporary discussions on sustainability and circular economy.

From an economic perspective, producers involved in both olive grove and vineyard management are uniquely positioned to leverage circular practices. This model facilitates cross-sector innovation, shared investment in sustainable technologies, and increased participation in certification programs to enhance competitiveness. Enterprises operating in both sectors can acquire, assimilate, and apply new knowledge essential for compliance with environmental regulations and meeting evolving consumer expectations. However, these transitions face structural barriers, including financial constraints, technological limitations, and the necessity for cultural and organizational changes [6]. Economically, the integration of wine and olive oil production provides resilience against market volatility. Collaboration between wineries and olive oil producers can extend tourist stays, distribute economic benefits more widely, and reinforce regional identity. Olive oil tourism, which is less developed than wine tourism, shares many structural similarities, including the type of visitors attracted, motivations for travel, and potential for regional revitalization. Tourists visiting wineries and oil mills are generally interested in the local culture, artisanal production, healthy food, and sensory experiences [7,8]. The similarities between these two types of tourism suggest strong potential for integration. Joint tourism strategies, such as shared promotional campaigns, coordinated itineraries, and unified branding, can increase demand, reduce seasonality, and improve profitability in both sectors. Furthermore, wine tourism has demonstrated the capacity to enhance environmental awareness, conserve rural landscapes, and support the maintenance of traditional agricultural practices [8,9]. Encouraging tourists to engage in both sectors not only strengthens the economic foundation of rural communities but also fosters a deeper appreciation for sustainable production systems.

Wine and olive oil, both essential elements of the Mediterranean diet, are renowned for their numerous health benefits, primarily owing to their rich polyphenolic content. These polyphenols possess significant antioxidant and anti-inflammatory properties, which contribute to the prevention of age-related diseases, particularly neurodegenerative and cardiovascular diseases. For example, compounds such as hydroxytyrosol in olive oil and resveratrol in wine have demonstrated neuroprotective effects by modulating mitochondrial function, reducing oxidative stress, promoting autophagy, and inhibiting the aggregation of amyloid beta and tau proteins, which are central to the pathology of Alzheimer’s disease [10]. In both preclinical and clinical settings, the consumption of these foods has been associated with improvements in cognitive performance, a reduction in biomarkers of neurodegeneration, and favorable modulation of brain metabolism and vascular function [10]. Moreover, olive oil and wine polyphenols have exhibited synergistic effects in enhancing insulin sensitivity and protecting against cerebral insulin resistance, which is another hallmark of neurodegenerative diseases [11]. The shared health-promoting potential of these two dietary liquids underscores their significance not only in culinary tradition but also in nutritional strategies aimed at disease prevention and healthy aging.

From an environmental perspective, the co-production of wine and olive oil provides valuable opportunities for circular economic practices. Both sectors generate substantial quantities of organic waste that can be repurposed, rather than discarded. Valorizing byproducts from both sectors supports resource efficiency and contributes to broader sustainability goals, particularly when these residues are used to develop new food products, natural preservatives, or other high-value goods. The environmental advantages of joint valorization strategies are apparent. By transforming organic waste into valuable inputs for food production, these initiatives can mitigate pollution, conserve resources, and extend the life cycle of agricultural materials. Concurrently, the adoption of such strategies draws upon a rich cultural and technical heritage. The Mediterranean region has historically sustained a food system predicated on the interdependence of wine and oil, and this legacy provides both inspiration and practical knowledge regarding contemporary sustainability challenges. Embracing this dual heritage through circular innovation may not only diminish environmental impacts but also reinforce regional identities and support more resilient agri-food economies [11,12,13]. Despite the mounting scientific interest in individual valorization pathways, few studies have adopted a joint approach that reflects the interlinked realities of production. This review was intentionally designed as a narrative review, which does not follow a predefined methodological protocol for literature search, screening, or selection, as is typical in systematic reviews. The narrative approach was chosen because it allows for the integration and critical interpretation of evidence from diverse domains, technological, nutritional, environmental, and regulatory, and is particularly suited to addressing broad and multidisciplinary questions. As argued in the literature, narrative reviews offer unique advantages such as providing in-depth critique, highlighting gaps and uncertainties in current knowledge, and identifying important unanswered questions, thereby helping to guide future research agendas and avoid redundant efforts [14,15]. In this context, the present review aims not to exhaustively catalogue all available studies, but rather to synthesize and critically discuss the main findings to date, while emphasizing the need for more integrated approaches to the valorization of olive oil and wine industry byproducts. In summary, this review aims to address this gap by critically examining the benefits, opportunities, and challenges associated with the transformation of wine and olive oil subproducts into food. Through this lens, we argue that their co-valorization can significantly enhance sustainability in Mediterranean agri-food systems, advancing innovation while preserving cultural heritage.

## 2. Olive Oil and Wine Industry Overview

### 2.1. Olive Oil

**Figure 1:**
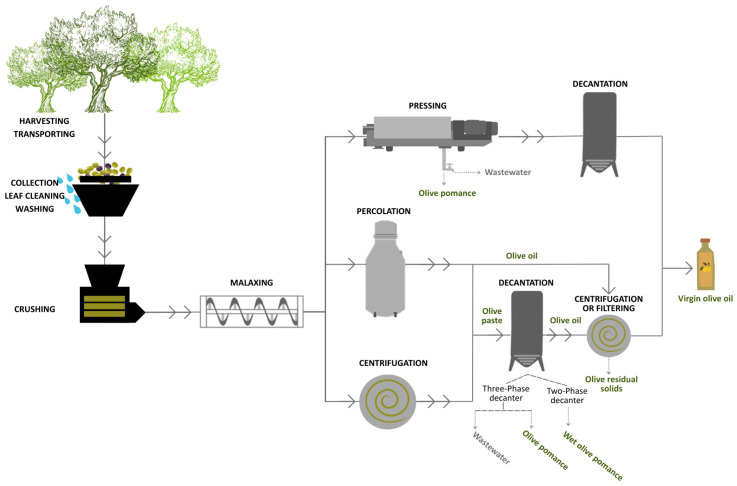
Flowchart of the virgin olive oil production process.

Olive oil production is a meticulous process that integrates traditional practices with modern technological advancements to ensure that the final product meets the desired quality standards. The workflow of olive oil is shown in Figure 1.

The process starts with the harvesting of olives, which are rarely handpicked in most producer countries, but are collected by means of several types of mechanical techniques, including branch vibrators and trunk-shaking tractors. These olives are transported to the oil mill with air circulation to prevent an increase in the temperature of the olives deposited at the bottom. Once they arrive, the olives are stored under optimal conditions until processing at the mill, which should be within the shortest possible time to avoid oxidation and retain low acidity [16,17].

The first step performed at the mill is the removal of foreign materials (e.g., leaves, sand, stones, etc.) and washing of the olives to remove impurities. Large solids are separated with the aid of water and mechanical methods, while light materials are removed mainly by a vibrating screen and air blowing [17,18]. Clean olives are then ground normally in semi-automated or completely automated modern olive mills to reduce their size until a uniform paste is obtained by breaking the pulp, skin, and stones. The crushing process is crucial to ensuring the final product’s sensorial quality, as well as the yield of the extraction process; thus, the type and speed of the crushers need to be modified to prevent scorching of the olive paste and reduce its propensity to oxidize [16,19]. The olive paste is transferred to a discontinuous step called malaxation, which involves the coalescence of tiny drops into larger ones and promotes the rupture of intact cells holding the oil. This is also a key stage because it induces substantial physical and biochemical transformations in olive paste, which are responsible for the final quality of the oil. In fact, temperature and time need to be controlled during this step because the extraction yield is directly correlated with these variables, as well as the presence or permanence of certain sensory or nutritional attributes [17,20,21].

After the malaxation step, the olive paste is composed of an oil phase, an aqueous phase, and insoluble solids that need to be separated. Three different extraction methods can be used: traditional pressing, selective percolation, and centrifugation. Traditional pressing, now largely obsolete, involves hydraulic pressure but tends to yield lower-quality oil [17,21]. Percolation, based on the lower interfacial tension of olive oil compared to water, allows high-quality oil recovery through stainless steel surfaces but is used less frequently [22]. Currently, centrifugation is the most widespread method, in the form of two-phase decanter systems, which separate oil and a wet pomace composed of solid and aqueous phases. This method minimizes water use and reduces environmentally harmful wastewater compared to the older three-phase system, which requires water addition and generates large volumes of olive mill wastewater. Although effective, this method generates large amounts of environmentally harmful wastewater [21]. Regardless of the method used for oil extraction, a final vertical centrifugation step with warm water is commonly applied to refine the virgin olive oil from the water and residual solid particles (e.g., broken olive stones), although is not always necessary [16,17].

Different types of waste are generated along the olive oil production line, although the most relevant are these associated with the separation stage. Byproducts generated during harvesting, such as leaves and twigs, are less significant but are sometimes considered together with other pruning residues (branches), since it is estimated that for every liter of olive oil produced, 6.23 kg of pruning residues (branches and leaves) are generated [23,24,25]. In terms of management, there is a lack of appropriate practices in Spain, which leads to the use of branches and leaves as firewood, while thinner remnants remain unexploited [26].

Regarding the residues generated in the mill, the solid waste olive pomace (OP) and wet olive pomace (OMSW) and olive mill wastewater (OMWW) can be distinguished, and their quantities largely depend on the extraction process applied (Table 1). Notably, the highest quantities of OMWW are generated by the three-phase centrifugation extraction method, whereas OMSW constitutes the primary residue resulting from the two-phase centrifugation extraction method. In the European Union, a Waste Framework Directive has been established (Directive 2008/98/EC) [27] that promotes the circular economy of diverse residues, including food waste. Member states are encouraged to prioritize waste prevention, reuse, and valorization over traditional disposal methods that promote research and innovation to enhance the sustainable use of olive residues. Spain transposed these principles into national legislation (Order TED/92/2022) [28], which established that olive residues can be considered byproducts when destined for crude OP oil extraction, although the EU framework allows for broader applications. Other important olive oil producers, such as Portugal and Italy, also have a legal framework for the management of olive mill waste that regulates the use of olive mill byproducts for agricultural and bioenergy applications [29].

### 2.2. Wine

**Figure 2:**
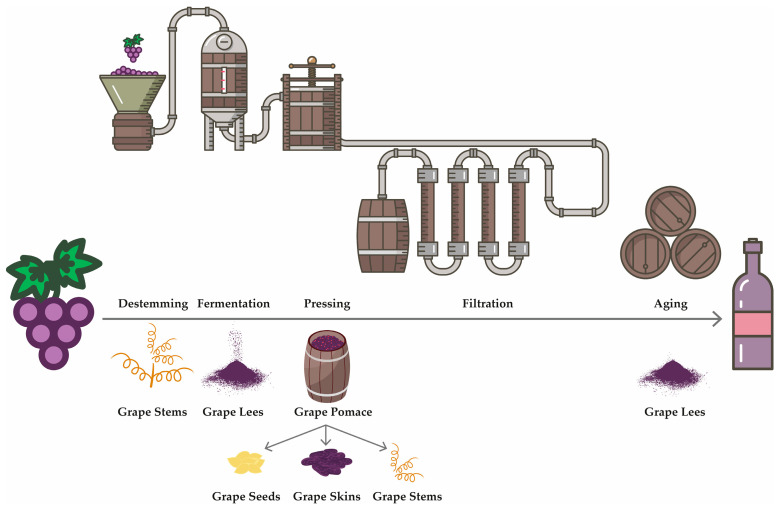
Overview of the winemaking process and main byproducts generated. The process begins with destemming, followed by fermentation, pressing, filtration, and aging, which results in bottled wine. Several byproducts are produced throughout the process: grape stems are removed during destemming, grape lees are formed after fermentation and again after aging, and grape pomace is generated during pressing, which includes grape seeds, skins, and residual stems. These byproducts represent potential resources for valorization in the food industry.

Vinification, the process of wine production, is a sophisticated biotechnological procedure that converts harvested grapes into wine through a sequence of meticulously regulated steps. Initially, the process involves harvesting grapes, followed by destemming and crushing to release the must (Figure 2). In white wine production, juice is separated from the skin prior to fermentation, whereas in red wine production, maceration facilitates extended contact between the juice and skin to extract color, tannins, and aromatic compounds. Alcoholic fermentation, predominantly driven by *Saccharomyces cerevisiae*, transforms sugars into ethanol and carbon dioxide and is often succeeded by malolactic fermentation to decrease acidity and enhance sensory characteristics. Subsequent clarification and stabilization processes eliminate suspended solids and prevent microbiological spoilage. The wine may then undergo aging, oxidation in wooden barrels, or reduction in bottles, depending on its intended style. Ultimately, the product is sterilized, bottled, and stored under controlled conditions [32,33]. Winery waste byproducts exhibit variability in composition and physicochemical properties contingent on the grape variety, vinification techniques, and processing conditions. The principal solid residues include grape pomace (comprising skins, seeds, and occasionally stalks), isolated grape seeds, skins, and stems (Figure 2). The primary liquid residue was winery wastewater. Each of these components has the potential for valorization, particularly in food and ingredient applications, owing to their bioactive compound content.

Wine production constitutes a significant agro-industrial activity globally and is intrinsically associated with the generation of substantial quantities of organic waste. According to recent data, global grape production has reached approximately 77.8 to 79 million tons of fresh grapes annually [34]. Of this total, an estimated 57%, equivalent to approximately 44.3 million tons, is allocated to winemaking processes, while the remainder is used for table grape consumption (approximately 36%) and raisin production (approximately 7%) [34]. Wine production results in the generation of significant quantities of both solid and liquid wastes, the characteristics and volume of which depend on the scale of production, grape variety, and specific winemaking methodologies [35]. In this paper, we exclusively focused on the waste streams generated from grape processing through winemaking, excluding those produced during vineyard cultivation (Table 2). The primary solid waste in wine production is grape pomace, a byproduct composed of grape skins, seeds, residual pulp, and occasionally stems, which is generated primarily during the crushing, pressing, and maceration stages. Grape pomace typically accounts for approximately 20–30% of the total grape weight, meaning that processing 1000 kg of grapes produces approximately 200–300 kg of pomace [36,37]. Based on the global volume of grapes used for wine production, the industry generates an estimated 8.9 to 13.3 million tons of grape pomace annually. Within this fraction, grape skins represent a substantial component, accounting for approximately 50–65% of pomace by weight [38]. Grape seeds constitute a major waste fraction, with global outputs estimated at approximately 3 million tons per year [39]. The stems, which are removed during the destemming process, account for approximately 3–7% of the total bunch weight, depending on the grape variety, number of berries per bunch, and sanitary conditions of the grapes [40]. Wine lees, which consist of yeast cells, tartaric acid crystals, and other precipitated solids, are generated during and after fermentation and stabilization. They typically represent 2–6% of the final wine volume and approximately 14% of the total solid waste generated during winemaking [41,42]. Wastewater is a major environmental concern in wineries. This liquid waste is generated in multiple stages, particularly during equipment washing, grape processing, fermentation, clarification, and bottling. Estimates suggest that the global wine industry produces between 650,000 m^3^ and 18,000,000 m^3^ of winery wastewater per year [43]. Winery wastes are characterized by low pH values (3.8–6.8), low electrical conductivity (1.62–6.15 dS/m), high organic matter content (669–920 g/kg or g/L), elevated concentrations of phenolic compounds (1.2–19.0 g/kg or g/L), and low levels of micronutrients and heavy metals, which not only pose environmental management challenges but also suggest a promising potential for valorization through applications such as biogas production, composting, or the extraction of bioactive compounds [44]. The diverse nature of winery waste underscores the importance of integrated waste management strategies, valorization approaches, and environmental safeguards to enhance the sustainability of wine production systems.

The effective management and use of winery waste constitute a crucial aspect of sustainability strategies within the wine industry. This waste is produced in substantial quantities and presents both environmental challenges and valuable resource opportunities. Their integration into circular economy models aligns with broader global sustainability goals and enhances the environmental and economic performance of wine production systems. From a circular economic perspective, winery waste valorization facilitates the transition from linear production models to closed-loop systems, in which waste is reconceptualized as a resource [43,45,46].

The high organic load and phenolic content of winery wastewater and solid residues make them suitable for composting and the extraction of high-value bioactive compounds, offering further avenues for economic valorization [13]. However, the regulatory framework surrounding winery waste management can significantly influence how these opportunities are realized. Current policies often favor large-scale, technologically advanced waste processing solutions that are more accessible to industrial producers and capable of absorbing high compliance and infrastructure costs. In contrast, small- and medium-sized producers face disproportionate financial and administrative burdens, particularly in relation to the mandatory distillation of pomace and logistical challenges of waste transport. Moreover, these regulations may restrict localized, small-scale circular practices, such as composting grape stalks or distilling pomace on-site, owing to concerns regarding standardization and market protection [47,48]. Despite these challenges, winery waste remains a promising avenue for improving sector sustainability. This reduces the environmental impact of viticulture and winemaking, lowers the costs associated with waste disposal, and creates new revenue streams through product diversification.

**Table 2:** Estimated quantities of major winery waste streams per 1000 kg of grapes processed, including composition, generation stages, and calculation assumptions.

Waste Type	Composition	Estimated Quantity	Stage of Generation	Reference
Grape Pomace	Skins, seeds, pulp, sometimes stems	200–300 kg (direct measurement: 20–30% of grape weight)	Crushing, pressing, maceration	[36,37]
Grape Seeds	Lignocellulosic matrix rich in lipids and polyphenols	30–60 kg (3–6% of fresh grape weight)	Pressing of grapes	[49]
Grape Skins	Cellulose-rich tissues containing pigments and polyphenols	100–150 kg (estimated as 50–65% of pomace; assuming pomace = 200–300 kg per 1000 kg of grapes)	Crushing and pressing	[38]
Grape Stems	Woody stalks from grape bunches	30–70 kg (3–7% of bunch weight; assumes bunch weight ≈ grape weight)	Destemming	[40]
Wine Lees	Yeast cells, tartaric acid crystals, other precipitates	16–48 kg (estimated from 2 to 6% of wine volume)	Fermentation, aging, stabilization	[41]
Winery Wastewater	Water with organics, acids, phenolics	375–10,500 kg (estimated from 500 to 14,000 L wastewater per 1000 L wine)	Washing, cleaning, fermentation, clarification, bottling	[43]

Notes: All estimated quantities are expressed in kilograms (kg) of waste generated per 1000 kg of processed grapes. Values derived from wine volume (e.g., wine lees and wastewater) were converted using standard assumptions: a grape-to-wine yield of 1000 kg grapes ≈ 750 L wine; lees density ≈ 0.8 kg/L; and wastewater density ≈ 1.0 kg/L. These conversions allow for consistent comparisons across waste types and highlight the relative contribution of each stream to the overall waste burden of winemaking.

## 3. Composition and Characteristics of Industry Wastes

The extraction techniques discussed in this review were selected based on their demonstrated efficiency, selectivity, and potential for sustainable application in the valorization of byproducts of the olive oil and wine industries. Methods such as ultrasound-assisted extraction and supercritical fluid extraction align with green chemistry principles by reducing solvent use, energy consumption, and environmental impact compared to conventional solvent-based approaches. Pressurized liquid and enzyme-assisted extractions offer improved yields of targeted bioactive compounds while maintaining the integrity of thermolabile molecules, making them suitable for functional food and nutraceutical applications. Furthermore, the scalability and food-grade compatibility of these techniques are critical for industrial implementation. By focusing on methods that combine environmental responsibility, extraction efficacy, and economic feasibility, this review highlights approaches that are most promising for advancing circular economy practices within the agri-food sector [50].

### 3.1. Olive Oil

Olive tree cultivation worldwide accounts for 25% of the total arable land, translating to 11.6 million hectares across 63 countries on five continents. According to the International Olive Council, global olive oil production has tripled over the last 60 years, reaching 2,564,000 t in the last crop year (2023/24), while the estimate for the 2024/25 crop year is 3,375,500 t (+32%), given that environmental conditions are favorable for olive oil tree cultivation [51].

Not all aspects or effects of olive oil production are positive. Extraction of olive oil from olives generates a significant amount of waste. When this waste is discharged without proper treatment, it poses a threat to the environment because of its toxicity and resistance to microbiological degradation [52].

The different processes for olive oil extraction mainly generate OP and OMWW residues and byproducts with a high content of valuable compounds. OP is a thick sludge that remains after the initial mechanical extraction of olive oil and is formed from olive skins, pulp, seeds, water, and stems [53]. Depending on the production line (two-phase or three-phase decanter), the pomace may contain water. A two-phase pomace has a moisture percentage close to 70%, whereas the moisture in the pomace of a three-phase system is approximately 45% [54,55]. In addition to water, cellulose, hemicellulose, and lignin in significant quantities [56], OP contains carbohydrates, lipids, peptides, flavonoids, phenolic compounds, and minerals [57].

On the other hand, OMWW is composed of soft tissues and water, and it contains carbohydrates, proteins, fatty acids, carotenoids, and a high phenolic content owing to its solubility in water. The most abundant phenolic compounds in OP and OMWW are hydroxytyrosol, tyrosol, oleuropein, and verbascoside, all of which have a wide array of biological values that are applicable in the pharmaceutical, cosmetic, and food industries [58,59]. Common methods for extracting these compounds include solvent extraction, supercritical fluid extraction, and membrane technology [60]. Advanced techniques such as ultrasound- and microwave-assisted extraction have also been explored [61].

Several extraction methods have been used to recover bioactive compounds from olive byproducts, each of which has specific advantages. Ultrasound-assisted extraction (UAE) uses ultrasonic waves to enhance mass transfer and improve extraction efficiency and is particularly effective for recovering phenolic compounds and triterpenes from olive leaves and olive pomace. Supercritical fluid extraction (SFE), which employs supercritical CO_2_ as a solvent, is especially suitable for extracting fatty acids and triterpenes while offering the benefit of being an environmentally friendly method. Pressurized liquid extraction (PLE), also known as accelerated solvent extraction, applies high temperatures and pressures to efficiently extract compounds, such as secoiridoids and flavonoids, from olive mill waste. Other green extraction techniques, including UAE, SFE, PLE, microwave-assisted extraction (MAE), and enzyme-assisted extraction (EAE), have been developed to minimize solvent use, reduce energy consumption and waste generation, and maximize bioactive recovery. Finally, chromatographic techniques such as high-performance liquid chromatography (HPLC) and gas chromatography coupled with mass spectrometry (GC–MS) are commonly used to identify and quantify the extracted compounds with high precision. These methods are chosen based on the specific bioactive compounds targeted and the type of olive oil waste being processed. For example, ultrasound-assisted extraction is highly effective against phenolic compounds, whereas supercritical fluid extraction is better for triterpenes and fatty acids (Table 3) [60,61].

Likewise, we must consider that the environmental conditions and the geolocation of olive crops also influence the amount of phenolic compounds. The composition of olive oil industry byproducts is susceptible to seasonal climatic conditions and geographical origin, which directly influence their potential for valorization in food and bioenergy applications. A four-year study in the Garda Bresciano region of Northern Italy analyzed olive oils from the “Casaliva” and “Leccino” cultivars and found that the total polyphenol content ranged from 150 to 350 mg/kg, depending on seasonal temperature and rainfall. In warmer years with higher heat summation during maturation (August–October), polyphenol levels were significantly higher. Similarly, tocopherol content varied from 180 to 260 mg/kg, with drier seasons favoring higher concentrations [62]. Geographically, a study comparing 19 Italian regions found that olive yield per hectare ranged from 1.2 to 4.8 tons, and irrigation water use varied from 0 to 3500 m^3^/ha, significantly affecting the environmental footprint and byproduct availability [63]. These differences also influence the concentration of bioactives in olive pomace and wastewater, which are key targets for valorization. In non-Mediterranean climates, olive oils have been found to have higher concentrations of β-tocopherol, ranging from 2 to 6 mg/kg, while α-tocopherol concentrations were below 250 mg/kg. In locations characterized by high temperatures, such as olive groves in Egypt, the α-tocopherol concentrations in oils can reach nearly 800 mg/kg [64].

### 3.2. Wine

Grape pomace is the main solid byproduct of winemaking and includes a heterogeneous mixture of skin, seeds, and occasionally stems. It exhibits high variability in composition but is generally rich in dietary fiber, lignin (32.5–56.7%), lipids (due to seed content), and a diverse phenolic profile, including protocatechuic acid, flavonols, anthocyanins, and tannins. The relative proportions of skin, seeds, and steams in pomace (~47%, 25%, and 28%, respectively) contribute to their complexity and multifunctional potential [36,65,66]. Comparatively, the phenolic profile of grape pomace is broader and more concentrated than that of other byproducts such as wine lees. While wine lees, the sediment formed after fermentation, contains phenolic compounds, including those adsorbed onto yeast cell walls, their total concentration is generally lower than that found in grape pomace. Moreover, grape pomace exhibits greater stability in terms of phenolic content and a richer composition of anthocyanins and tannins [36,67]. The lipid content of pomace is significantly influenced by the presence of seeds, which are a source of polyunsaturated fatty acids, such as linoleic acid [68,69]. In addition to phenolic compounds, grape pomace contains structural proteins and essential minerals such as iron, calcium, zinc, and potassium, thereby enhancing its nutritional and functional value [70,71]. Grape seeds, commonly recovered from pomace during winemaking, are rich in bioactive compounds, particularly polyphenols such as proanthocyanidins. These compounds contribute to their high antioxidant potential, making grape seeds attractive for application in functional foods and nutraceuticals. Their composition includes carbohydrates, proteins, and lipids, with reported values of approximately 40% fiber, 16% oil, 11% protein, and 7% complex phenolics, such as tannins, in addition to sugars and mineral salts [72]. Grape skins are primarily lignocellulosic in nature, consisting of cellulose, hemicellulose, and lignin, and are a major source of anthocyanins, flavonoids, and phenolic acids, particularly in the red grape varieties. These compounds are of interest because of their antioxidant, anti-inflammatory, and coloring properties in food systems [73,74,75]. Grape stems, removed during the de-stemming process, are rich in dietary fiber and are largely composed of structural carbohydrates such as cellulose, hemicellulose, and lignin. They also contain phenolic compounds that can be extracted for use as natural antioxidants or antimicrobial agents [76,77]. Variations in the biochemical composition and structure of winemaking residues influence their extraction efficiency and suitability for diverse applications. Winery wastewater is generated during various stages of winemaking, notably during cleaning, fermentation, and bottling. It is characterized by low pH, high organic load, and the presence of phenolic compounds, which pose environmental management challenges but also offer opportunities for recovery and reuse [78]. Wine lees are generated as byproducts at various stages of the winemaking process, predominantly during alcoholic and malolactic fermentation. The composition of wine lees includes dead yeast or bacteria contingent on the wine type, along with their metabolites, phenolic compounds, tartaric salts, and plant material derived from grapes. Phenolic compounds, primarily originating from grapes, are absorbed by microorganisms via biotechnological processes. Wine lees are important in the food industry because of their substantial content of bioactive compounds, notably phenolic compounds and anthocyanins, which have antioxidant and antimicrobial properties [79]. Notably, grape pomace is distinguished among winery byproducts because of its abundant and diverse bioactive content, high fiber and oil yields, and favorable extraction potential [80]. Nevertheless, stalks, skins, seeds, lees, and winery wastewater also present unique advantages for biotechnological valorization [81]. The development of integrated waste-recovery strategies, particularly those aligned with circular economic principles, is crucial for improving the sustainability and economic performance of wine production. 

## 4. Bioactive Compounds in Olive Oil and Wine Industry Wastes

### 4.1. Factors Affecting Stability and Interactions of Bioactive Compounds

The stability and interactions of bioactive compounds derived from olive oil and wine byproducts are significantly influenced by processing parameters, such as temperature, time, and pH, both during extraction and subsequent food applications. Understanding these mechanisms is crucial for optimizing yield and preserving bioactivity. For instance, high temperatures and prolonged extraction times can lead to the degradation of thermolabile phenolic compounds, such as anthocyanins, hydroxytyrosol, and other flavonoids, via oxidation, hydrolysis, and polymerization reactions [82,83]. In the case of grape pomace, extraction at temperatures exceeding 60 °C or durations beyond 30 min have been shown to decrease the anthocyanin yield by up to 50%, whereas maintaining temperatures below 50 °C and employing shorter times can mitigate these losses [82,83]. Similarly, hydroxytyrosol and secoiridoids in olive oil byproducts exhibit greater stability when extracted under mild thermal conditions, as these compounds are sensitive to heat-induced degradation [84]. pH also plays a critical role, as phenolic compounds are generally more stable under acidic to neutral conditions (pH 3–7), whereas alkaline environments promote hydrolysis and oxidative browning, altering their structure and bioactivity [82]. In the case of anthocyanins from grape skins, the flavylium cation form predominates at low pH and is more stable and intensely colored, whereas higher pH values lead to color loss and structural degradation [83]. Additionally, interactions between bioactive compounds and the food matrix, such as binding to proteins or starch, can affect extractability, stability, and sensory properties. The enzymatic activity (endogenous or added during processing) can also modify the chemical structure of phenolic compounds, potentially enhancing or diminishing their bioavailability and bioactivity [84]. These mechanistic insights underscore the importance of optimizing the process conditions, such as temperature, time, pH, and matrix interactions, to maximize the recovery and functionality of bioactive compounds, ensuring that their beneficial effects are retained in the final food products.

### 4.2. Olive Oil

The key bioactive compounds mainly found in olive oil waste are hydroxytyrosol, tyrosol, oleuropein, and verbascoside, each with unique chemical properties [60]. There are different factors upon which the quantity and variety of these bioactive compounds depend, such as the harvest time, olive variety, and cultivar, as well as the processing and extraction processes [85]. These compounds are integral to the chemical composition of olive oil and contribute to its stability, flavor, and potential health benefits. Their diverse structures and solubility profiles make them versatile for various applications, from food preservation to pharmaceuticals.

Hydroxytyrosol (C_8_H_10_O_3_) is the main product of oleuropein hydrolysis and is responsible for various varieties of olive oil and its flavors [85]. This phenylethanoid, with a benzene ring substituted by two hydroxyl groups and an ethyl group, is highly soluble in water and other polar solvents, making it easily absorbable [86].

Tyrosol (C_8_H_10_O_2_) is another phenylethanoid structurally similar to hydroxytyrosol, but with one hydroxyl group. It is a colorless solid with a melting point of 91–92 °C and is primarily found in olive oil. Oleuropein (C_25_H_32_O_13_) is a glycosylated secoiridoid that consists of a glucose molecule linked to hydroxytyrosol and elenolic acid. It is known for its bitter taste and is removed during the processing of olives to make them edible. Oleuropein is slightly soluble in dimethyl sulfoxide (DMSO), ethanol, and methanol. Verbascoside (C_29_H_36_O_15_), also known as acteoside, is a polyphenol glycoside that combines hydroxytyrosol and caffeic acid moieties bound to disaccharides (rhamnose and glucose). It is a white to pale yellow solid with a melting point of 232 °C and is slightly soluble in dimethyl sulfoxide and methanol [4].

Bioactive compounds from olive oil waste have been linked to various health benefits such as anti-inflammatory, antioxidant, and anticancer properties [87]. For example, hydroxytyrosol inhibits low-density lipoprotein (LDL) oxidation, which is beneficial for cardiovascular health [61]. However, not only are there health benefits of hydroxytyrosol, as it protects against oxidative stress and inflammation, it is also effective against various pathogens, shows potential in inhibiting cancer cell growth, preventing skin damage and aging, and osteoporosis, and has been described as a potential protective agent against HIV infection and related diseases [61].

Tyrosol plays an important role in muscle protection by inhibiting muscle damage. Furthermore, this compound can regulate cholesterol efflux and protect intestinal mucosa. It also helps in managing diabetes, preventing hypertension, protecting against heart disease, and preventing osteopenia [61].

Oleuropein has strong antioxidant properties, lowers blood pressure, protects against heart disease, inhibits cancer cell growth, and protects nerve cells and skin from damage. It has been described as anti-diabetic and anti-obesity and can protect the liver and stomach lining. This compound also prevents osteoporosis [61].

Finally, verbascoside protects against oxidative stress and inflammation and shows potential in inhibiting cancer cell growth, protecting the liver, promoting wound healing, lowering blood pressure, protecting nerve cells, helping in managing diabetes, and protecting against UV damage [61].

### 4.3. Wine

The biological effects of polyphenolic compounds derived from winemaking byproducts have been extensively investigated and reviewed in the scientific literature. This section succinctly summarizes these effects, emphasizing their biological potential and underscoring their significance in the food industry. Winery waste, particularly grape pomace, seeds, skins, lees, stems, and pomace, is increasingly being acknowledged as a valuable source of bioactive compounds with diverse health-promoting properties. Table 4 provide a comparative overview of the extraction methods, total polyphenol yields, and main polyphenol classes identified in the different byproducts of the grape and wine industry. While these data serve as a reference for understanding the potential of each waste stream, it is important to note that both the quantity and composition of polyphenols can vary significantly depending on multiple factors, including grape variety, cultivation practices, vinification processes, storage conditions, and specific parameters of the extraction method used. Polyphenols are the most abundant of these compounds and have been extensively studied. Polyphenols are a broad class of secondary metabolites, including flavonoids (anthocyanins, flavanols, and flavonols), phenolic acids, stilbenes (resveratrol), and tannins [38,88,89,90,91]. These polyphenols are distributed across all winery byproducts, including grape skins, stems, and seeds, and their concentrations are influenced by grape variety, growing conditions, and winemaking processes [91]. In addition to polyphenols, winery waste contains dietary fiber, both soluble and insoluble, as well as organic acids, minerals, and fatty components, particularly grape seeds. Grape seed oil is rich in polyunsaturated fatty acids such as linoleic acid, tocopherols (vitamin E), and phytosterols, all of which contribute to its nutritional and therapeutic potential [49,92,93]. These bioactive compounds have multiple health benefits, particularly those related to oxidative stress and inflammation. The antioxidant capacity of winery byproducts has been consistently reported in in vitro and in vivo studies, including protection against lipid peroxidation, increased endogenous antioxidant enzyme activity, and free radical scavenging [38,88,89,94]. Anti-inflammatory properties have also been observed, with polyphenols reducing the production of pro-inflammatory cytokines and modulating inflammatory pathways, which may be relevant for chronic disease prevention and management [38,91]. Numerous studies have highlighted the cardiovascular benefits of these compounds. Grape-derived polyphenols have been shown to reduce LDL oxidation, enhance endothelial function, lower blood pressure, and regulate platelet aggregation, thereby collectively contributing to cardiovascular protection [49,88,90]. At the metabolic level, compounds from grape pomace and stems exhibit anti-diabetic effects, including improved insulin sensitivity, inhibition of carbohydrate-digesting enzymes, and regulation of blood glucose levels [89,90,91].

Their anticancer effects have also been extensively documented. Polyphenols from winery waste can modulate pathways involved in cell proliferation and apoptosis, such as the cyclooxygenase-2 (COX-2), epidermal growth factor receptor (EGFR), and estrogen receptor pathways. These compounds have demonstrated cytotoxicity against various cancer cell lines, including breast, colon, skin, and liver [38,89,93]. Winery waste extracts have the capacity to protect against oxidative stress-induced DNA damage, further supporting their potential role in cancer prevention. Polyphenols from grape byproducts can mitigate oxidative DNA damage through their antioxidant activity, which involves scavenging free radicals, enhancing antioxidant enzyme activities, and modulating signaling pathways related to oxidative stress responses. Specifically, grape pomace extract has been shown to reduce oxidative DNA damage in colon cancer cells, while polyphenols such as resveratrol have additional protective effects owing to their well-documented antioxidant properties [96].

Another promising research area is the study of gut health. Polyphenols and dietary fibers from grape byproducts can modulate the gut microbiota composition, enhance intestinal barrier function, and promote the production of short-chain fatty acids (SCFAs), which play crucial roles in digestive and systemic health [91,93]. These prebiotic-like effects are associated with reduced inflammation, improved lipid metabolism, and potentially lower risks of obesity and metabolic syndromes [89,97].

Additional bioactivities encompass antimicrobial properties, evidenced against both Gram-positive and Gram-negative pathogens, and potential neuroprotective effects attributed to anthocyanins and resveratrol [38,49,90]. Furthermore, certain constituents of wine lees and grape skins demonstrate anti-aging and skin-protective effects through the inhibition of elastase and tyrosinase [38,79]. Collectively, this evidence highlights the diverse bioactivities of winery waste components and their prospective applications in the nutraceutical, functional food, and pharmaceutical industries. Nonetheless, although the in vitro and preclinical findings are promising, further human clinical trials are essential to substantiate these effects and optimize their application in health-promoting products.

## 5. Applications in Food Industry

### 5.1. Olive Oil

Over the past two decades, the production and consumption of olive oil have increased worldwide owing to its organoleptic and health properties. It can be said that it has become a trendy food. Additionally, consumers increasingly demand healthier foods; therefore, the industry and scientific community have started to develop new functional ingredients for food and beverages. However, we do not forget that some of this waste can also be used in animal feed. The Olive Feed Corporation has developed a proprietary process to transform olive waste into commercially viable animal feed, addressing issues of digestibility, palatability, and safety [98]. The incorporation of olive soap stocks increases the energy value of the swine diet, resulting in sausages with reduced lipid oxidation, while preserving flavor, texture, color, and nutritional value [99]. Supplementation with byproducts of OMWW reduces oxidative stress in the plasma and tissues of broiler chickens, resulting in higher-quality meat [100]. In the field of human nutrition, enriched foods containing byproducts from olive waste are also consumed. The supplementation of wheat pasta with polyphenols from OP enhances fiber content and firmness and decreases cooking time [101]. The shelf life of bakery products and meat can be increased by fortifying them with polyphenols from OMWW, thereby decreasing microbial growth [102,103]. The fortification of other cereal-based products, such as bread and pasta, with OP and OMWW has improved their chemical quality and antioxidant activity, with OP showing greater phenolic enrichment. However, it negatively impacts the sensory properties due to bitterness [104]. It has been proposed to cure food in extra-virgin olive oil for phenolic enrichment. Castillo-Luna and Priego-Capote [105] demonstrated that curation enhances foods’ antioxidant capacity and offers a viable preservation strategy to improve health benefits. Specifically, the oleuropein aglycone concentration in fish and cheese was higher at the beginning, whereas in the studied vegetables, it increased until day 16. Hydroxytyrosol increased during the second half of the curation period and reached comparable levels in fish, vegetables, and cheeses, while tyrosol followed the same trend in fish and cheese, showing a more complex dynamic in vegetables.

In this review, we specifically focus on which beverages and foods have been supplemented with the main polyphenols obtained from OP and OMWW (hydroxytyrosol, tyrosol, oleuropein, and verbascoside) and their beneficial effects.

In line with the ongoing market trend associated with the increasing demand for natural products, bioactive compounds from olive byproducts, especially hydroxytyrosol, have been proposed as natural preservatives based not only on their antimicrobial and antioxidant properties but also on their ability to remain active for a longer time than other phenolic compounds [106]. This was observed when polyphenol extracts enriched with hydroxytyrosol were incorporated into refined oil, resulting in better preservation of the α-tocopherol content compared to synthetic antioxidants such as butylated hydroxytoluene [107]. Similarly, phenolic compounds from olive byproducts have been added to sunflower oil to enhance oxidative stability, an effect primarily linked to the presence of hydroxytyrosol and oleuropein [108]. Furthermore, their application in the preservation of edible oils has been supported by the use of immobilized esterase catalysis to modify functional food products with hydroxytyrosol, which has shown promise in enhancing oxidative stability and reducing rancidity [109].

The replacement of sulfur dioxide by hydroxytyrosol in red and white wine induced an intensification of color, scents, and taste at bottling. Color improvement was observed even after six months of storage in white wines [110]. Moreover, its supplementation provided an additional antioxidant effect during the first six months of storage in red organic wines. Those enriched with 120 mg/750 mL of hydroxytyrosol were initially preferred for their sensory attributes, while those with lower concentrations (30 mg/750 mL) were better appreciated after 9 and 12 months of storage [111].

Another fermented beverage, beer, has been supplemented with olive extract to enhance its antioxidant activity and color, particularly at higher concentrations, not surpassing 1%, since foam stability can be affected [112]. The addition of hydroxytyrosol to beer during the boiling phase of brewing results in a softer and more pleasant flavor and aroma as it alters bitterness and enhances antioxidant activity [113].

Some researchers have explored the possibility of creating functional beverages enriched with hydroxytyrosol to improve blood flow and enhance the body’s antioxidant capacity, while maintaining the original smell and flavor. Fuwa et al. formulated functional beverages composed of hydroxytyrosol, caffeine, glucose, and maltose with a pleasant taste and sweetness [114]. Tomato juice and soap enriched with this polyphenol have been formulated to improve the antioxidant activity of consumers [115]. Moreover, the combination of coffee with OP showed an increase in the phenolic content and antioxidant capacity of the beverage, with the 10% OP blend exhibiting the highest bioactivity and sensory acceptance by mice, whereas a 200 µg/mL dose resulted in 30.5% α-amylase inhibition [116].

The addition of hydroxytyrosol to yogurts preserved their organoleptic properties, improved their antioxidant activity and the growth of lactic acid bacteria, and contributed to preventing spoilage during fermentation. This, in turn, helped to decrease LDL cholesterol, body weight, and blood pressure [117]. Hydroxytyrosol has also been used to increase antioxidant activity and decrease lipid and protein oxidation in meat and fishery products, sausages, and patties, while preserving color, flavor, and texture. Moreover, this supplementation allows for the preservation of these foods against microorganisms and fungi, thereby extending their shelf life [118,119,120]. In bakery products, fortified biscuits with hydroxytyrosol darkened the color and improved the antioxidant activity in 13 volunteers who consumed 30 g of biscuits supplemented with 5.25 mg of hydroxytyrosol, resulting in reduced levels of oxidized LDL in their blood [121].

Tyrosol has several health benefits [122,123]. However, its hydrophilic nature limits its use in the food industry. They can interact with proteins and lipids, which may alter the texture, stability, and sensory characteristics of the final product. Therefore, tyrosol has been microencapsulated for use as a dietary supplement, which enhances its bioaccessibility to the body [124].

Oleuropein has been used as a bioactive ingredient in functional foods with improved properties. The enrichment of pasteurized milk with 7.75 mg oleuropein/100 mL milk increased its shelf life by 60%, and its incorporation in yogurts provided higher firmness and viscosity, and less syneresis [125]. The incorporation of oleuropein into meat burgers exerts antioxidant effects, increasing the shelf life of the product. In salmon burgers, lipid oxidation is reduced during storage at low temperature [126].

Verbascoside has multiple beneficial effects on organisms, acting as a hepatoprotective, anti-diabetic, anti-inflammatory, and neuroprotective agent [127]. Thus far, its use in the food industry has been limited. It has been demonstrated that the incorporation of verbascoside in fermented sausages reduces fungal growth without modifying sensory characteristics, and in fresh meat, causes significant bacterial death, slows the spoilage rate, and increases shelf life [128,129].

### 5.2. Wine

#### 5.2.1. Grape Pomace

Grape pomace has emerged as a versatile wine byproduct that is incorporated into a diverse array of food matrices. Foods such as bread, pasta, dairy products, baked goods, chocolates, beverages, and processed items have been fortified with grape pomace to enhance their antioxidant content, dietary fiber, and overall nutritional value. These formulations not only mitigate food industry waste but also align with consumer trends favoring health-promoting and functional foods [13].

The integration of grape pomace-derived ingredients into fermented dairy products enhances their nutritional, functional, and sensory properties. In probiotic goat milk, grape pomace extracts have been found to increase the total phenolic content and antioxidant capacity, while preserving the viability of probiotic strains, particularly *Lactobacillus acidophilus*, although *Lactobacillus rhamnosus* exhibited greater stability during storage. Products combining *Lactobacillus rhamnosus* and grape pomace not only maintained superior flavor, color, and acceptability over a 28-day period but also exhibited slight improvements in firmness and consistency without compromising cohesiveness or viscosity [130]. In fermented skim milk, extracts from grape cultivars, such as Pinot Noir, accelerated acidification and supported probiotic strains, such as *L. acidophilus* and *Streptococcus thermophilus*. The phenolic compounds in the extracts can be metabolized by these bacteria, potentially enhancing their health benefits. The phenolic composition of the extract and its interactions with specific probiotics are crucial for optimizing the product performance [131]. In yogurt, the addition of wine grape pomace increased fiber content and antioxidant activity while reducing lipid oxidation. Although higher concentrations initially resulted in lower viscosity and increased syneresis, these textural issues diminished over time. The sensory panels preferred yogurts with 1% pomace, which achieved an optimal balance between health benefits and consumer appeal [132]. In cheese, particularly fresh ovine cheese, grape pomace enhances protein content, reduces fat, and increases antioxidant capacity. Although it altered the color (more red, less bright) and volatile compound profiles, it did not inhibit starter culture growth. Cheeses with grape pomace are described as being more aromatic and acidic, with increased fiber content, friability, and perceived humidity, all contributing to their sensory acceptance and supporting the development of functional cheeses using winemaking byproducts [133,134]. These studies indicate that grape pomace, whether in the extract or powder form, is a promising and sustainable ingredient for enhancing the health value of fermented dairy products. Although formulation-specific adjustments may be necessary, their benefits for nutritional quality, probiotic support, and sensory attributes are well-substantiated.

The integration of grape pomace into chocolate formulations constitutes a promising approach to augmenting nutritional value, particularly by enhancing polyphenol content and antioxidant capacity. In milk chocolate, which generally contains fewer cocoa-derived antioxidants than dark chocolate, the addition of grape pomace, either as whole pomace or grape seed flour, has been demonstrated to significantly increase total polyphenol levels and antioxidative potential without diminishing sensory appeal. Chocolates incorporating moderate amounts of grape pomace (5–7.5%) not only maintained acceptable rheological and textural properties but also received higher overall sensory ratings compared to traditional milk chocolate. Importantly, the fruity note imparted by the pomace positively influenced flavor perception, whereas slight darkening in color did not negatively impact consumer evaluation. However, inclusion levels near 10% resulted in an increased particle size and reduced smoothness, adversely affecting the texture. These findings highlight the necessity of achieving a particle size below 30 µm to preserve the desired mouthfeel and prevent grittiness [135]. Processing parameters, particularly the timing of pomace incorporation, are also crucial in determining the final product’s quality. When grape pomace was added early in the production process, it was more uniformly coated with fat, leading to improved flow properties. Conversely, late-stage addition increases flow resistance owing to inadequate fat dispersion on the pomace particles [135]. Similar outcomes have been observed in the development of chocolate spreads, in which dried grape pomace was used to partially replace sugar- and milk-derived powders. This substitution enhanced the total phenolic and resveratrol content, contributing to the functional profile of the product. However, higher concentrations of pomace were associated with reduced spreadability and increased firmness. Sensory analyses indicated that formulations containing 3–5 g of grape pomace per 100 g achieved the most favorable balance between nutritional enhancement and sensory acceptability [136]. These studies illustrate that grape pomace can be effectively utilized as a functional ingredient in chocolate products, supporting health-related claims, while preserving or enhancing sensory characteristics. Optimal results depend on controlled particle size, appropriate processing techniques, and moderate inclusion levels to mitigate potential negative effects on texture and spreadability.

Grape pomace has also gained recognition as a functional and sustainable ingredient in meat and fish products, enhancing their nutritional value, technological performance, and safety. Grape pomace stabilized through low-temperature drying and fine milling provides dietary fibers that enhance the water-holding capacity, texture, and color of processed meats and fish. Inclusion levels of 1–2% are generally effective in delivering benefits without adversely affecting the sensory properties [137]. In salmon burgers, the incorporation of grape pomace flour significantly increased dietary fiber content and delayed lipid oxidation during frozen storage without compromising microbiological or nutritional quality. Although the product’s visual appearance was altered, consumer acceptance remained favorable [138]. Similarly, beef derived from steer-fed dried grape pomace exhibited improved oxidative stability during storage, with reductions in lipid and protein degradation, and the maintenance of desirable color characteristics [139]. Pork and poultry products also benefit from grape pomace supplementation. In pork burgers, grape pomace extract enhances lipid stability and color retention without influencing microbial growth. Extracts with higher polyphenol contents demonstrated superior antioxidant activity, indicating a dose-dependent relationship [140]. Moreover, in beef and chicken meatballs, the use of grape seed extracts substantially decreased the formation of heterocyclic aromatic amines and other mutagenic compounds generated during high-temperature cooking methods such as charcoal grilling and oven roasting. This effect is attributed to the polyphenol-rich profile of the grape seed extract, which disrupts free-radical pathways in the Maillard reaction [141]. Collectively, these findings support the integration of grape pomace and its derivatives into meat and fish products as a strategy to improve shelf life, nutritional profile, and food safety while aligning with clean-label and sustainability goals. The success of such applications depends on the form, polyphenol content, and concentration of the grape pomace used, as well as the processing conditions and the specific product matrix.

The composition and antioxidant potential of grape pomace flours from various cultivars and their blends were examined to determine their relevance to sustainable diets and food innovation. Grape pomace flour, a byproduct of the wine and grape juice industries, offers a promising avenue for reducing waste while contributing to nutrient-enriched, functional food ingredients. The analysis revealed variations in phenolic profiles, nitrogen compounds, and antioxidant capacities across cultivars and formulations, highlighting the importance of cultivar selection and blending to optimize nutritional benefits. Red grapes such as “Máximo,” “Bordo,” and “Violeta” demonstrated superior anthocyanin content and antioxidant capacity, reinforcing their potential as sources of natural antioxidants and colorants. “Niagara Rosada” exhibited lower levels of bioactive compounds; however, when incorporated in blends up to 25%, it maintained high antioxidant activity while reducing allergenicity and enhancing levels of rutin and caffeic acid. This finding is relevant for the development of safer and more inclusive food products. The quantification of phenolic compounds, amino acids, and biogenic amines provided evidence of their nutritional and functional value. These bioactive-rich flours are viable alternatives to synthetic additives and offer applications as natural preservatives, colorants, and supplements. Their incorporation into food systems could promote sustainable dietary patterns by valorizing waste streams and minimizing reliance on non-renewable resources [142].

The incorporation of grape pomace into cereal-based foods presents a promising strategy for enhancing nutritional value while promoting sustainable food systems. As a byproduct of winemaking, grape pomace is abundant in polyphenols and dietary fiber, and its use can improve the health profile of bread and other similar products. Numerous studies have demonstrated its capacity to augment antioxidant levels and fiber content; however, preserving the technological and sensory qualities that are crucial for consumer acceptance remains a challenge. The inclusion of grape pomace powder in wheat bread formulations has been shown to significantly elevate total dietary fiber and phenolic compounds while enhancing antioxidant capacity. Nevertheless, these enhancements are accompanied by alterations in physical characteristics such as reduced loaf volume, increased crumb density, and darker coloration. The extent of these changes is contingent upon the type and concentration of pomace utilized. Breads containing 5–10% grape pomace exhibited markedly higher nutritional values, but also showed diminished loaf volume and altered texture, particularly at higher inclusion levels [143,144]. The increased water-binding capacity of the fiber appeared to disrupt gas retention during fermentation, resulting in denser structures. Despite these sensory challenges, certain cultivars have demonstrated a better performance in terms of consumer acceptance. Breads enriched with Cabernet Sauvignon pomace maintained an acceptability comparable to that of white bread, whereas others, such as Muscadine Noble, negatively affected the aroma and texture [143]. Similarly, the addition of grape pomace from “Zelen” and “Merlot” cultivars affected the crumb texture, with “Merlot” presenting greater sensory challenges, such as increased sandiness and aftertaste, even as it contributed more to the antioxidant profile [145]. These cultivar-dependent effects underscore the necessity to tailor formulations based on the desired balance between health benefits and sensory appeal. Grape pomace powder has been shown to reduce starch hydrolysis and the predicted glycemic index of wheat bread, indicating its potential benefits for metabolic health. The bioaccessibility of phenolic compounds, particularly anthocyanins, is maintained at significant levels post-digestion, highlighting the role of these fortified products in modulating oxidative stress and glycemic responses [146]. Notably, these functional benefits were achieved without significantly affecting overall sensory acceptability when the inclusion levels were restricted to 5 or 10%. Beyond bread, the incorporation of grape pomace in breadsticks also resulted in nutritional enhancements, with increases in the total phenolic content and antioxidant capacity. However, similar to bread, these benefits are accompanied by structural and sensory modifications. Breadsticks fortified with grape pomace exhibit reduced volume, increased acidity, wine odor, and astringency [147]. Nevertheless, they maintained good overall sensory acceptability, suggesting that consumers may tolerate or even appreciate certain flavor changes associated with perceived health benefits. A more concerning finding emerged from the shelf-life studies. Although grape pomace fortification increased the antioxidant capacity, it unexpectedly reduced the oxidative stability of the breadsticks during storage. Thermodynamic analysis revealed that oxidation was endergonic and non-spontaneous, yet proceeded more rapidly in the presence of pomace, possibly due to the higher polyunsaturated fatty acid content or pro-oxidant behavior of certain phenolic compounds. These results underscore the complexity of food matrix interactions and the necessity for further refinement of storage conditions and formulation strategies [148]. Despite the variations in texture, appearance, and sensory attributes, all studies have concluded that grape pomace is a valuable ingredient for sustainable food reformulation. It promotes a circular economy by converting agricultural waste into nutrient-dense food components, and its incorporation into cereal-based products aligns with strategies to enhance dietary quality while reducing environmental impact. However, careful selection of the grape variety, particle size, and inclusion level remains crucial to ensure technological functionality and consumer acceptance. Overall, although technological and sensory trade-offs are nontrivial, the consistent nutritional advantages of grape pomace across different formulations (bread, breadsticks, and other cereal-based goods) affirm its role as a multifunctional, sustainable food ingredient. Continuous innovation in processing techniques and consumer communication is essential to fully unlock its potential to advance sustainable dietary patterns [143,144,145,146,147,148].

The integration of grape pomace into baked goods presents compelling evidence of its potential to enhance nutritional quality while promoting environmentally sustainable food systems. Cakes, muffins, biscuits, and jelly candies fortified with grape pomace consistently exhibit improved nutritional profiles. Multiple studies have reported increased levels of total dietary fiber, total phenolic content, anthocyanins, and antioxidant activity in grape pomace-enriched products compared to conventional formulations [149,150,151]. These components are associated with a reduced risk of chronic diseases and enhanced digestive health, indicating the potential of grape pomace to contribute significantly to sustainable diets that prioritize nutrient density and plant-based ingredients [152]. Beyond compositional enrichment, several studies have emphasized that the bioactivity of grape pomace components, such as α-glucosidase and pancreatic lipase inhibition, could aid in regulating glycemia and lipid metabolism, particularly in products with higher levels of pomace inclusion [152]. Furthermore, one serving of certain grape pomace-supplemented baked goods has been shown to meet or exceed the daily recommended intake of anthocyanins and phenolic compounds, underscoring their relevance in functional food development [153]. Despite its nutritional benefits, the incorporation of grape pomace affects the texture, structure, and sensory acceptance of grape products. The literature indicates that increased fiber content due to pomace addition alters product moisture, hardness, and cohesiveness. Cakes and muffins enriched with grape pomace become denser and darker and exhibit greater chewiness, particularly at higher inclusion levels [149,150]. Muffins with coarser pomace particles demonstrate better volume, a more uniform crumb structure, and greater overall acceptability, whereas finer particles result in increased astringency, crumb stickiness, and irregular pore formation [150]. Optimal inclusion levels vary by product type but generally range from 4 to 15%. Cakes with 4% grape pomace received the highest sensory ratings, balancing improved nutrition with acceptable texture and flavor [149]. Gluten-free muffins containing 15% grape pomace are preferred because of their sweet taste and moist texture, whereas higher levels produce gritty textures and overly intense flavors [154]. In jelly candies, grape pomace enhances antioxidant content and sensory appeal when particle sizes are tailored to optimize texture and flavor release [151]. Biscuits have emerged as particularly successful matrices for the incorporation of grape pomace. Studies using Malbec and Tannat grape pomace have reported high acceptability, even at 20% inclusion, with improvements in flavor and bioactivity, including enhanced antioxidant capacity and enzyme inhibition, associated with metabolic health benefits [152,153]. In these cases, pomace addition either maintains or improves textural qualities and consumer perception, suggesting that the product type and processing methods critically shape sensory outcomes. High concentrations of grape pomace can compromise the product quality, particularly in terms of texture and consumer acceptance. Sensory attributes, such as bitterness, astringency, and grit, become more pronounced at elevated inclusion levels, necessitating the careful formulation and selection of ingredients. Moreover, the pomace particle size plays a significant role in determining both the nutritional and sensory characteristics of the final product, indicating the need for standardized processing protocols tailored to each food category [150,151]. The integration of grape pomace with baked goods is a nutritionally and environmentally beneficial innovation that contributes to sustainable food systems. When thoughtfully formulated, grape pomace-enriched products offer a means to enhance fiber and antioxidant intake while supporting waste reduction in the winemaking sector. Further research on consumer education, processing optimization, and long-term health impacts is essential to fully realize the potential of this underutilized resource.

Research has consistently indicated that the enrichment of pasta with grape pomace or grape pomace flour enhances its fiber content, antioxidant activity, and levels of bioactive compounds such as polyphenols, flavonoids, tocochromanols, and carotenoids. These compounds, which are retained even after cooking, contribute to reducing the predicted glycemic index of the final product and improving its nutritional value [144,155,156]. Notably, the use of whole grape pomace, encompassing skins, seeds, and stems, further amplifies the diversity and potency of bioactive compounds in enriched pasta [155]. From a technological standpoint, the incorporation of grape pomace influences the cooking properties of pasta. Most formulations exhibit increased cooking loss and an extended optimal cooking time, which is attributed to alterations in the gluten–starch network caused by high fiber and polyphenol contents [156,157]. Despite these modifications, cooking loss in enriched pasta generally remains within acceptable limits, particularly at low inclusion rates such as 5–10% [155,158]. Sensory evaluation yielded mixed outcomes. Although high concentrations of grape pomace may adversely affect flavor, aroma, and texture, moderate additions, especially around 5%, tend to preserve or even enhance consumer acceptability, particularly after cooking [157,158]. Its appearance is also affected, with enriched pasta often acquiring a purple or reddish hue, which may be perceived positively or negatively depending on the formulation and target market [156,159]. The use of grape pomace significantly contributes to the circular economy by redirecting winemaking residues from waste streams into the food chain. This practice aligns with sustainability objectives and offers economic benefits by generating value-added products from low-cost input [160,161]. Furthermore, the combination of grape pomace with other byproducts, such as OP, can enhance the health-promoting properties of pasta while diversifying the phenolic profiles and fiber types [161]. Nonetheless, some challenges persist. At higher levels of incorporation, the sensory attributes and structural properties may deteriorate. Interactions between polyphenols and gluten proteins can weaken the protein network, thereby affecting the texture and protein retention [157]. Additionally, cooking can significantly reduce phenolic content, although soluble phenols may increase, potentially offsetting some losses in antioxidant activity [155,159]. Future innovations, including optimized extraction techniques and emerging green technologies, present opportunities to enhance the retention and bioavailability of bioactive compounds in functional pasta [158,160]. These advancements could mitigate the quality drawbacks while preserving the health and environmental benefits of grape pomace fortification. In conclusion, grape pomace-enriched pasta has considerable potential as a functional food, which aligns with the principles of sustainable nutrition. When carefully formulated, such products can meet consumer expectations while delivering enhanced health benefits and reducing food waste. Further research into consumer preferences, processing techniques, and long-term health impacts is essential to fully integrate these products into sustainable dietary patterns.

It is worth noting that the relatively more extensive discussion on grape pomace compared to other byproducts, such as wine lees, reflects the larger number of original research articles available on grape pomace. This is consistent with the fact that grape pomace is the most abundant byproduct of the wine industry and has, therefore, attracted greater interest from producers and researchers over a longer period. Consequently, the scientific literature on grape pomace is more developed, justifying the broader coverage of this topic in the present review.

#### 5.2.2. Grape Seed Oil

The integration of grape seed oil and grape pomace into various food products has gained increasing attention owing to their nutritional benefits and potential contributions to sustainability [13]. Grape seed oil derived from winery waste has been effectively used in multiple food applications including yogurt, chocolate, cocoa spreads, sausages, and canned fish. Its incorporation can enhance the nutritional profile of foods, extend shelf life, and modify certain sensory properties, although the outcomes vary significantly depending on the product type and concentration [49].

The integration of grape seed oil into yogurt formulations could potentially contribute to more sustainable diets, primarily through nutritional enhancement and shelf-life extension. Grape seed oil is abundant in beneficial fatty acids and exhibits high oxidative stability owing to its fatty acid composition, particularly its stearic (28.70%) and palmitic (7.88%) acid contents, rendering it a valuable ingredient for culinary applications that require thermal resilience and nutritional value [162]. When incorporated into yogurt at varying concentrations (1.5, 2.5, and 3.5%), it influenced key quality parameters during refrigerated storage. Notably, the 3.5% formulation exhibited the lowest peroxide values after 14 days, indicating reduced lipid oxidation and enhanced oxidative stability, which are essential for extending shelf life and reducing food waste [162]. Furthermore, the absence of *Escherichia coli* throughout storage underscores microbiological safety. Although lactic acid bacteria initially increased and then declined, this pattern remained within the acceptable limits, supporting the hygienic viability of grape seed oil-enriched products. From textural and compositional perspectives, oil increases yogurt viscosity, improves water-holding capacity, and reduces syneresis, all of which are desirable for enhancing consumer experience and product consistency [163]. These improvements also suggest a reduced need for additives, aligned with the clean-label trends. Although yogurts with higher oil concentrations (3%) exhibited greater firmness over time, which was linked to increased total solids and protein interactions, the most favorable sensory profile was observed in yogurts with 1.5% oil. This low concentration preserves nutritional benefits while maintaining consumer acceptance, particularly in terms of appearance and mouthfeel. However, flavor acceptability was higher in controls because of the mild but perceptible aroma of grape seed oil, indicating a sensory trade-off at higher inclusion levels [163]. These studies highlight the potential of grape seed oil to enhance both the nutritional profile and stability of yogurt, with a 1.5% concentration offering the best compromise among health benefits, technological functionality, and consumer acceptability.

The incorporation of grape seed oil into chocolate-based products presents significant nutritional and functional advantages pertinent to sustainable dietary strategies; however, it poses considerable challenges in terms of sensory acceptability and product consistency. In hybrid oleogel/hydrogel chocolate systems, grape seed oil has been shown to enhance the overall sensory ratings. However, optimal formulation ratios remain undefined, thereby limiting their immediate applicability to product reformulation in an acceptable manner to consumers [164]. More critically, substituting refined sunflower oil with cold-pressed grape seed oil in cocoa spreads, particularly when combined with 10–15% encapsulated grape seed extract, substantially increased the polyphenol and flavonoid content, thereby enhancing the antioxidant capacity and improving the nutritional profile of the spread [165]. From a sustainable diet perspective, this substitution is promising, as it encourages the use of byproducts (grape seeds) and replaces resource-intensive oils with a functional alternative. Nonetheless, these nutritional benefits are accompanied by sensory drawbacks, such as increased hardness, viscosity, and undesirable changes in mouthfeel (notably greater graininess and reduced shine), along with a diminished sweet profile and intensified grape seed aroma, all of which adversely affect consumer acceptance [164,165]. Overall, while grape seed oil contributes to functional and nutritional enhancements in confectionery products, advancing objectives related to nutrient density and the valorization of food industry byproducts, its inclusion must be carefully optimized. These studies underscore the tension between health promotion and sensory quality, highlighting the importance of formulation strategies that reconcile functionality with palatability to render healthier options viable for sustainable diets.

The use of grape seed oil in the preservation of fish, encompassing both canned and refrigerated products, presents promising nutritional and functional benefits consistent with sustainable dietary principles. However, significant challenges must be addressed before its widespread adoption. In the context of sardine canning, grape seed oil surpasses olive oil in terms of polyphenol, flavonoid, and polyunsaturated fatty acid content, particularly linoleic acid, thereby enhancing the nutritional profile of the preserved product [166]. Sardines canned in grape seed oil show elevated levels of fat, protein, and ash and show improvements in lipid quality markers, such as an increased polyunsaturated-to-saturated fatty acid ratio (>2) and reduced atherogenic and thrombogenic indices. These alterations suggest a potential reduction in the risk of cardiovascular disease, underscoring the functional value of grape seed oil as a preservation medium. Notably, lipid oxidation and protein degradation remained minimal over time, indicating that the antioxidant properties of grape seed oil effectively maintained product stability. Nonetheless, while the linoleic acid content increased, a decline in eicosapentaenoic acid, a vital omega-3 fatty acid, was observed, indicating a possible nutritional compromise that warrants further investigation. Additionally, the absence of sensory evaluation of sardines preserved in grape seed oil limits the assessment of consumer acceptance, which is a crucial factor for sustainable dietary transitions [166]. Beyond thermal preservation, the application of grape seed oil in oil-in-water nanoemulsions for chilled flat-headed mullet fillets has demonstrated strong preservative effects when combined with cinnamon essential oil [167]. These nanoemulsions decelerated spoilage indicators, such as pH shifts, total volatile base nitrogen, and lipid oxidation, and extended the sensory shelf life from 10 to 14 days. They also enhanced the textural and olfactory attributes, indicating their potential as natural antimicrobial and antioxidant agents. This aligns with the shift away from synthetic additives and supports cleaner-label aquatic products [167]. Nevertheless, critical issues including cost-effectiveness, consumer perception, allergenicity, environmental implications, and scalability remain unexplored. Overall, the integration of grape seed oil into fish preservation offers nutritional enhancement and food waste reduction, which are key pillars of sustainable diets [166,167]. However, gaps in long-term performance data, sensory profiling, and feasibility analysis highlight the need for comprehensive evaluation before grape seed oil can be considered a viable large-scale alternative for aquatic food systems.

Grape seed oil has been investigated as a component of vegetable oil blends to partially substitute animal fat in meat products, such as emulsion-type pork sausages and frankfurters. In sausage formulations, it is used at concentrations of 2–4%, along with other oils, such as olives and canola. These mixtures enhance the water-holding capacity and decrease hardness and chewiness, likely due to their high content of unsaturated fatty acids. Grape seed oil, which contains 58–78% linoleic acid, contributes to a higher proportion of polyunsaturated fatty acids and a reduced saturated fat content in the final product [168]. Grape seed oil has been evaluated as a fat substitute in frankfurters at levels ranging from 1% to 10%. It modifies the color of the product, rendering it lighter and more yellow, while diminishing redness at concentrations exceeding 4%. These visual changes result from the naturally lighter color of grape seed oil than that of animal fat. However, these visual enhancements did not increase the sensory acceptance. Frankfurters with grape seed oil consistently received lower scores in taste and overall acceptability, primarily because of the persistent aroma and flavor of the unrefined oil. Nevertheless, among grape seed-derived ingredients (extract, oil, and flour), oil ranked second in acceptability, indicating that its potential can be enhanced by improved refinement and deodorization [169]. Although grape seed oil increases the risk of lipid oxidation owing to its high unsaturated fat content, oxidation levels remain below spoilage thresholds for over 90 days, demonstrating that shelf life is not compromised. These findings underscore a trade-off: while grape seed oil enhances the nutritional profile of meat products, it may adversely affect flavor and consumer acceptance unless properly processed. Further research is warranted to refine the oil and to determine the optimal inclusion levels that preserve both health benefits and sensory quality [168,169].

The incorporation of grape seed flour into bread production has emerged as a promising strategy to enhance the nutritional profile of bakery products while addressing the sustainability challenges of agro-industrial waste management. Grape seed flour is derived from grape pomace, is a byproduct of winemaking, and is rich in phenolic compounds and dietary fiber. One study demonstrated that increasing the concentration of grape seed flour led to significant improvements in total phenolic content, a marker associated with potential health benefits, such as antioxidant activity [170]. However, the enhancement of nutritional value has resulted in considerable trade-offs. As the proportion of grape seed flour increased, the key physical attributes of bread, including loaf volume and brightness, decreased, while firmness and porosity increased. These changes negatively affected consumer acceptance, particularly at substitution levels of 7.5% and above. Notably, bitterness and astringency became less acceptable to consumers at the highest inclusion levels, indicating that taste remains a significant barrier to the incorporation of high levels of grape seed flour [170]. Complementary findings were reported in a sensory analysis study that compared direct and indirect methods of bread preparation using up to 9% grape seed flour [171]. The results highlight the complexity of optimizing both the technological and sensory parameters. Although higher percentages of grape seed flour consistently intensified crumb and crust coloration and introduced distinct taste profiles characterized by increased bitterness and sourness, the most acceptable formulation was identified at 5% replacement using the indirect method. This approach produced a bread product with sensory characteristics most similar to traditional black bread, including improved crumb softness and flavor persistence. Additionally, the indirect method resulted in more uniform crumb pore structures and slightly higher scores for flavor and aroma intensity, suggesting that processing techniques play a crucial role in moderating the sensory impact of grape seed flour [171]. Storage conditions, particularly freezing, also influence the quality and acceptability of grape seed flour-enriched breads. Although the total phenolic content remains stable regardless of the frozen storage time, the structural and sensory properties are affected [170]. Loaf volume and cell diameter decreased, while firmness increased, especially after six weeks of frozen storage. These changes were more detrimental at higher replacement levels, exacerbating diminished consumer acceptability. Nonetheless, at a moderate level of 5 g grape seed flour per 100 g wheat flour, the negative impact of freezing was relatively minor, suggesting that this formulation may strike a feasible balance between nutrition, sustainability, and consumer satisfaction. Collectively, these studies revealed that while grape seed flour fortification offers clear nutritional and environmental benefits, its successful integration into bread products depends on maintaining sensory qualities that are acceptable to consumers. Both the amount of grape seed flour used and breadmaking method significantly influenced the outcomes. A substitution level of approximately 5% appears to be a critical threshold for maintaining a desirable sensory profile while delivering measurable increases in fiber and phenolic contents. These findings are essential for guiding food innovation to support sustainable diets. They illustrate how upcycling a winery byproduct can contribute to reducing food system waste while offering functional benefits, provided that consumer preferences are carefully considered during product development [170,171].

#### 5.2.3. Grape Skins

The integration of grape pomace skins into muffin recipes enhances their dietary fiber content and promotes the sustainable utilization of winemaking byproducts. Bender et al. [172] used grape skin flour from Riesling and Tannat varieties to substitute wheat flour at levels of 5, 7.5, and 10% in muffin formulations. This study assessed the impact of dietary fiber content, technological characteristics, and sensory attributes. The inclusion of grape skin flour increased the total dietary fiber content, particularly that of the soluble fraction. All enriched formulations met the criteria to be labeled as high in fiber, supporting their potential as functional foods. From a technological perspective, grape skin flour influences quality parameters. The muffins showed a darker color in the crumb and crust, increased hardness at higher inclusion levels, and decreased cohesiveness. These changes were attributed to reduced air incorporation during mixing, increased density, and alterations in water absorption and gel formation due to lower protein and starch content from reduced wheat flour. Chewiness increased across the formulations, which was consistent with the increased hardness. Springiness was not significantly affected by grape skin flour, and resilience was decreased in most cases, except for the 5% Tannat skin flour sample. Despite textural changes, the sensory evaluations showed that consumer perceptions were largely unaffected. For muffins with tannin skin flour, participants reported no significant differences across sensory attributes, including texture, and instrumental changes did not translate into noticeable differences among consumers. In muffins with Riesling skin flour, texture acceptability declined at 7.5% inclusion, although the 5 and 10% formulations were better received. This suggests that moderate adjustments to the grape skin-flour percentage can maintain favorable sensory profiles. Purchase intention corroborated these findings, as muffins with 5 and 10% grape skin flours from both varieties received the highest evaluations. Although male participants gave higher scores for aroma and flavor in the Riesling-based muffins, no gender differences emerged for texture, indicating consistent texture acceptability across demographics. The analysis showed that, in Tannat-based muffins, color received the lowest acceptability score, although this was not statistically significant. In Riesling-based muffins, the texture and color varied, particularly at mid-level inclusions. This study demonstrated that grape pomace skins from Riesling and Tannat grapes can effectively enhance the nutritional value of muffins without compromising their sensory quality. The results showed the potential of grape skin flour as a functional ingredient that adds value to winemaking byproducts while supporting the development of bakery products with improved fiber content and acceptable technological and sensory properties [172].

The integration of grape pomace skin from winemaking into pasta formulations has been investigated. In the work of Gaita et al. [173], grape pomace skins from Pinot Noir and Italian Riesling grapes were used to partially substitute wheat flour in pasta at concentrations of 3, 6, and 9%. This study evaluated the effects of these byproducts on the antioxidant properties, phenolic content, and sensory attributes of pasta. The analysis identified polyphenolic compounds in pasta samples enriched in grape pomace skins, including gallic, ferulic, coumaric, rosmarinic, caffeic, epicatechin, rutin, quercetin, kaempferol, and resveratrol. Higher percentages of grape pomace skin corresponded to an increased total phenolic and antioxidant capacity. The inclusion of Italian Riesling grape skins resulted in a greater enhancement of these values compared to Pinot Noir, likely because of variations in winemaking. Unlike Pinot Noir pomace, which underwent fermentation, Italian Riesling pomace was not fermented with juice. This difference may have preserved more beneficial compounds in Italian Riesling grape skins. In the sensory evaluation, pasta samples with up to 6% grape pomace skins showed enhanced characteristics compared to the control, particularly Italian Riesling grape skins. The best sensory outcomes were observed in pasta containing 3% Italian Riesling grape pomace, followed by samples with 6% of the same variety and those with 3 and 6% Pinot Noir pomace. These improvements were attributed to the formulation of grape pomace skins, wheat flour, and eggs without the addition of water. This composition helped to preserve the structural integrity of pasta during processing and boiling. Although the study did not specify individual sensory attributes, it reported that enriched pasta samples were rated “very good” compared to the control’s “good” rating. Although color changes were not explicitly quantified, inferences could be made based on grape varieties. Pinot Noir contains anthocyanins that impart reddish hues, whereas Italian Riesling lacks these pigments. Thus, pasta with Pinot Noir pomace likely developed a darker coloration, whereas that made with Italian Riesling showed subtle changes. Differences in pomace processing, such as fermentation in Pinot Noir but not in Italian Riesling, could have influenced pigment retention and color transfer to pasta. Higher pomace concentrations may have intensified these color differences. At 9% substitution, the pasta formulations encountered processing difficulties, indicating a limit to pomace incorporation without compromising manufacturability. Despite this limitation, this study demonstrated the potential of repurposing winery byproducts in functional foods. By enhancing pasta’s nutritional and sensory qualities while addressing winery waste concerns, this research supports efforts to align food innovation with sustainability goals [173].

#### 5.2.4. Wine Lees

The incorporation of wine lees, a byproduct of winemaking, into food products has attracted growing interest because of its potential to enhance nutritional profiles while promoting sustainability. Research on foods enriched with wine lees demonstrates not only significant improvements in functional and health-promoting properties but also offers promising pathways for circular economy practices within the food industry. In yogurt formulations, the inclusion of fine wine lees from Cabernet Sauvignon significantly enhanced the nutritional and functional properties of the product [174]. Increasing both the concentration of wine lees and the duration of absorption resulted in higher levels of proteins, carbohydrates, anthocyanins, phenols, and antioxidant activity. These enhancements were accompanied by improved rheological characteristics such as increased water-holding capacity and reduced syneresis, indicating enhanced product stability and texture. Notably, the highest concentration tested (16% wine lees with a three-hour absorption) yielded the most pronounced improvements. These findings underscore the dual benefit of repurposing winery byproducts to create value-added dairy products that align with consumer demand for health-focused foods, while mitigating environmental waste. Similar benefits were observed when wine lees flour was used to partially replace wheat flour in biscuits [175]. The fortified biscuits, particularly those with 20% substitution, exhibited an increased fiber and protein content, elevated phenolic compounds, and significantly higher antioxidant activity. These nutritional gains were accompanied by a reduced glycemic index and starch hydrolysis rate, suggesting that biscuits can help moderate postprandial glucose responses, which is an important feature in the context of metabolic health. Moreover, the prebiotic potential of the fortified biscuits, evidenced by an increased density of lactic acid bacteria, points to added gut health benefits. However, the formulation changes introduced by wine lees also result in altered sensory characteristics including increased acidity, bitterness, and astringency. While these shifts may reduce appeal among traditional consumers, they also offer opportunities for segmentation in health-conscious and novelty-seeking markets. The enhanced oxidative stability observed in the fortified biscuits suggests an extended shelf life, although a higher moisture content may require careful storage to prevent spoilage. In baked goods, such as muffins, wine lees have demonstrated efficacy as a fat substitute, achieving reductions in calorie content while enhancing dietary fiber [176]. At substitution levels ranging from 25 to 50%, muffins retained acceptable technological and sensory attributes; however, higher substitution levels resulted in increased hardness, chewiness, and acidity, accompanied by a distinct wine-like taste and odor. These alterations may appeal to specific consumer segments but also indicate the need for further formulation optimization to ensure broader market acceptance. Notably, the incorporation of wine lees in this context not only supports healthier formulations by reducing fat content but also exemplifies responsible resource utilization by transforming winery waste into a functional ingredient. Across yogurt, biscuits, and muffins, the use of wine lees revealed a consistent pattern of enhanced nutritional quality, improved functional properties, and added sustainability benefits, which were often counterbalanced by significant changes in sensory attributes. These studies collectively underscore the importance of targeted product development and consumer education in facilitating the successful adoption of wine lees-based products. From a sustainability perspective, integrating wine lees into food production represents a strategic approach to reducing agro-industrial waste while contributing to the development of functional foods that promote healthier and more environmentally responsible diets.

## 6. Challenges

### 6.1. Olive Oil

Cost-effectiveness, effective extraction, recovery, and processing, as well as overcoming market limitations, are the primary technical and logistical obstacles to using bioactive compounds from olive byproducts [177]. These are complex matrices with a high moisture content, which makes it difficult to selectively extract high-value compounds without substantial energy costs or extensive extraction and purification steps [178]. In this context, certain bioactive constituents such as oleuropein and hydroxytyrosol are chemically unstable and prone to oxidation, thereby requiring controlled extraction and storage conditions that may not be optimal for achieving higher yields [177,179]. Furthermore, the concentrations of valuable phenolic and triterpene substances that are potentially extractable from olive byproducts are often low, making their recovery and purification challenging in terms of achieving a commercially viable yield [18,180].

While bioactive compounds in olive byproducts can generate diverse high-value products for the food industry, commercial feasibility is limited by high investment costs, limited subsidies, and logistical challenges. Although some innovative business models exist, broader success requires stronger public–private collaboration, policy incentives, research partnerships, and support mechanisms to scale up technologies and market acceptance [178]. Related to the latter, the main barrier is consumer skepticism towards the production technologies involved in the process rather than the products themselves. Gaining broader acceptance requires targeted marketing strategies, and consumer education focuses on clear communication between the health and environmental benefits of these bioactive components [181].

Furthermore, the valorization of these compounds in the food industry faces several regulatory hurdles. Directive 2008/98/EC does not clearly define whether they should be classified as waste, byproducts, or food ingredients, leading to significant legal and operational uncertainties [27]. In addition, obtaining authorization as a novel food under Regulation (EU) 2015/2283, along with meeting the stringent requirements of the European Food Safety Authority (EFSA) for health claims under Regulation (EC) No. 1924/2006, involves a complex regulatory process. To date, hydroxytyrosol remains the only authorized bioactive compound from olive byproducts and has received novel food authorization [182,183,184]. This is also the case of regulations in Australia and New Zealand, where a specific concentration of hydroxytyrosol (20 mg/g) extracted from olive fruit is considered a novel substance and no safety concerns have been established by Food Standards Australia New Zealand (FSANZ) [185]. By contrast, in the United States, hydroxytyrosol is generally recognized as safe (GRAS status), both synthetic and olive-derived, and more broadly used across foods, beverages and supplements with fewer restrictions [186]. In China, hydroxytyrosol has been classified as a food additive by the National Health Commission, primarily for its antioxidant properties, although this is limited to hydroxytyrosol synthesized through fermentation using *Corynebacterium glutamicum*. Hence, hydroxytyrosol extracted from olive byproducts cannot legally be used in food products [187]. These differences between regulatory frameworks highlight the significant obstacles that bioactive compounds extracted from olive byproducts face in important markets which could not only limit their global market potential but also discourage investment in their industrial scale. These limitations also provide a starting point for further research and innovation. Priority areas include the development of green and scalable extraction technologies such as supercritical fluids, ultrasound, and microwaves, which may enhance the yield, selectivity, and environmental sustainability of the process. In this context, supercritical CO_2_ has already offered an environmentally friendly alternative to conventional solvents for the extraction of antioxidant-rich lipid fractions, showing potential scalability when combined with photovoltaic energy sources [188]. Moreover, other supercritical fluid-based techniques have demonstrated the efficient encapsulation and incorporation of olive leaf bioactive compounds into polymers, offering stable antioxidant delivery systems [189,190]. Although scaling up requires further optimization to maintain uniformity and functionality, recent studies have shown positive results. A pilot-scale integrated valorization that extracted 1500 t/y of olive pomace through polyphenol recovery and energy self-sufficiency has demonstrated economic feasibility with an NPV of EUR 1.99 million and 58% IRR. However, this study highlights the hydroxytyrosol extract price and sales volume as key profitability drivers [191].

The application of omics techniques in this field is of interest for further development. Metabolomics is a powerful tool for characterizing the complex chemical profiles of olive oil byproducts, allowing for the better identification of bioactive compounds. Metabolomics guides data-driven decisions to maximize the yield and quality of bioactive compounds [192,193]. One of the most interesting end-use applications in the food industry is the development of functional foods and nutraceuticals enriched with bioactive compounds, which can be incorporated into a variety of food matrices. Nevertheless, a key research priority is to guarantee chemical stability, bioavailability, and consumer acceptability. The development of encapsulation technology, customized extraction techniques, and in vivo bioactivity evaluation are crucial [159,194]. Furthermore, achieving regulatory harmonization is essential to ensure product quality, safety, and consumer trust across all industries, with the potential application of these compounds.

### 6.2. Wine

Despite the growing interest in valorizing winery byproducts for their bioactive potential, the transition from laboratory-scale extraction to industrial application remains fraught with challenges. A central issue is the scalability of extraction techniques such as microwave-assisted and supercritical fluid extraction, which, although environmentally promising, require significant energy input, solvent recovery infrastructure, and specialized expertise, rendering them financially inaccessible for many small and medium-sized producers [36,195]. These economic constraints are further compounded by the seasonal nature of grape pomace production and the compositional variability of winery residues, which are influenced by grape variety, terroir, and vinification methods. Such heterogeneity complicates the development of standardized extraction protocols and undermines batch-to-batch reproducibility, particularly for compounds such as anthocyanins and flavonoids, which are highly sensitive to pH and oxidation [36,196]. Unlike lipid-rich matrices such as olive paste, winery byproducts such as grape pomace and wastewater are highly fibrous or aqueous, necessitating distinct extraction approaches. For instance, membrane-based separation is often employed in wastewater valorization; however, its efficiency is heavily dependent on the choice of the membrane material and finely tuned operating conditions. Factors such as pressure, temperature, and pH significantly influence the rejection of phenolic compounds, whereas membrane durability and fouling potential affect the operational sustainability [95]. Although materials such as cellulose acetate are cost-effective, their instability under acidic or thermal conditions limits their long-term application. More robust membranes, such as polyimide or polyvinylidene fluoride, offer greater resistance, but increase complexity and cost [95]. Furthermore, grape-derived bioactive compounds are particularly susceptible to thermal degradation and oxidative reactions, particularly during prolonged processing or solvent exposure. In contrast to hydroxytyrosol from olives, which exhibits greater stability in certain lipid environments, grape-derived polyphenols require stringent control of environmental conditions to maintain their functionality for application in food [36,195]. In this context, cold extraction and encapsulation strategies show promise but remain under-optimized for many grape-specific compounds. Nonetheless, some studies have highlighted the scalability and simplicity of certain valorization processes, making them economically promising. The use of hot water as the sole solvent for extracting bioactive compounds from grape pomace under food-grade conditions has proven to be both environmentally friendly and technically scalable to pre-industrial levels (1:10 scale) [197]. In a comparative study, Constantin et al. [198] confirmed the scalability of subcritical water extraction in clean-label product applications. Crucially, a scale-up study about the extraction of high-added-value grape pomace using supercritical fluid from 1 L to 500 L vessels was able to maintain the manufacturing cost of EUR 67/kg of extract [199]. Altogether, these findings show a promising scenario since the proven scalability and practical design of these processes make them a realistic opportunity to move toward more sustainable and profitable production. Regulatory and market barriers impede progress. Although many of the same legal ambiguities affecting olive byproducts also apply, particularly concerning the classification of these substances as waste, byproducts, or food-grade ingredients, such as grape pomace and winery wastewater, they often face additional scrutiny due to microbial contamination risks and fermentation byproducts. The approval process for functional claims or food use is lengthy, costly, and often prohibitive for small enterprises [195]. For example, while dry grape extract from grape pomace is authorized by the EU as an animal feed additive under Regulation (EC) No 1831/2003, its use in human food remains unregulated, and purified grape phenolics (e.g., resveratrol, anthocyanins…etc.) may undergo novel food regulation when used outside traditional matrices and have only been considered as valid candidates when chemically synthesized [200]. Outside the EU, regulatory approaches to bioactive compounds derived from winery byproducts also vary considerably. Several grape-derived ingredients, such as grape seed extract and grape pomace extract, have received the GRAS status in the United States, allowing their incorporation into foods, beverages, and supplements without prior market approval [201]. In contrast, China lacks a dedicated regulatory category for most grape byproduct extracts used in food [202]. As in the case of bioactive compounds derived from olive oil byproducts, the lack of harmonized or product-specific regulations limits international trade and investment in this sector. Moreover, despite the potential to integrate grape bioactives into diverse consumer products, limited public awareness and inconsistent environmental and health benefits hinder market uptake. Critically, economic evaluations of winery waste valorization remain scarce, leaving producers without robust models for forecasting investment returns. Although proposals for shared infrastructure, cooperatives, and academic-industry partnerships are gaining traction, they are not yet widely implemented or supported by targeted incentives [195]. Addressing these bottlenecks will require not only technological innovation but also systemic shifts in policy, funding mechanisms, and cross-sector coordination. To unlock the full potential of winery byproducts within a sustainable food system, future research must prioritize scalable and cost-effective extraction methods tailored to grape-specific matrices, along with regulatory harmonization and value chain integration.

The integration of grape pomace into cereal-based foods such as pasta, bread, noodles, crackers, and baked goods presents a significant potential for enhancing nutritional profiles and promoting sustainable diets. However, this process is accompanied by technical and logistical challenges that may compromise product quality, consistency, and consumer acceptance. These challenges are not only specific to the formulation but are also significantly influenced by the origin, composition, and physicochemical properties of grape pomace. The most notable obstacle is the adverse effect on the rheological and structural integrity of doughs. High levels of grape pomace, particularly exceeding 6%, substantially hinder gluten network formation, resulting in decreased elasticity, reduced extensibility, and increased tenacity in various dough systems [145,147,160,173]. This compromised structure often leads to an inferior volume, increased cooking loss, and textural inconsistencies in the final product [156,203]. For instance, in pasta, the incorporation of 9% grape pomace skin results in poor kneading behavior and difficulty in shaping, ultimately leading to a statistically significant decline in sensory quality [173]. These technological limitations restrict the maximum usable concentration of grape pomace and undermine the feasibility of its widespread application in cereal-based foods. Color changes represent another significant challenge. Grape pomace, particularly red varieties, impart intense hues ranging from violet to deep brown depending on the food matrix and grape variety used [155,204]. While these changes may be marketed as indicators of added nutritional value, they can also deter consumers from becoming accustomed to conventional visual profiles. Furthermore, sensory modifications such as increased bitterness, astringency, and acidity often accompany pomace inclusion, especially at higher concentrations, thereby reducing consumer acceptance [152,159,170]. Challenges in processing also pertain to the stability of the bioactive compounds, which is the primary rationale for utilizing grape pomace. Phenolic compounds, such as anthocyanins and flavanols, are prone to degradation during the baking and drying processes. For instance, in baked goods, approximately 84% of phenolics may be lost during processing [152,153]. Although acylated anthocyanins demonstrate greater heat stability than their non-acylated counterparts, significant losses remain a concern, thereby questioning the functional benefits of the final product [153]. Similarly, cooking pasta and noodles often results in increased cooking loss and reduced firmness, accompanied by a decreased antioxidant potential and nutrient retention [155,203]. Variations in grape pomace composition owing to varietal differences, geographical origin, and vinification processes further complicate the formulation [155,160]. These variations affect not only the nutritional properties but also the behavior of pomace during processing, making batch-to-batch standardization challenging without advanced quality control protocols. In particular, particle size plays a critical role in the texture, hydration, and integration into dough systems. Larger particles are associated with poor cohesiveness and increased cooking losses, whereas micronization enhances adhesiveness, starch digestibility, and water absorption, thereby improving both technological and nutritional profiles [150,159]. To address these technical barriers, emerging processing strategies have been proposed. These include hydration techniques, particle size optimization, and the incorporation of quality enhancers, such as transglutaminase or carboxymethylcellulose, to improve structural integrity and sensory properties [159]. Advanced methods such as microencapsulation and green extraction technologies, including ultrasound-assisted or supercritical fluid extraction, offer potential pathways for preserving and delivering bioactive compounds more effectively [160]. However, these approaches require significant investment and further validation, which limits their current scalability and practical application in conventional food production. Importantly, despite the nutritional advantages such as increased fiber content, phenolic content, and antioxidant activity, there remains a critical need to balance these gains against the decline in technological and sensory performance. Products fortified with grape pomace have the potential to qualify for high-fiber labeling and appeal to health-conscious consumers [144,204]. However, widespread adoption will depend not only on optimizing formulation and processing but also on effective consumer communication and education. Consumer acceptance may hinge just as much on perceived health benefits such as taste, appearance, and texture [173,203]. In summary, the valorization of grape pomace in cereal-based products represents a sustainable and nutritionally meaningful intervention; however, it is not without considerable technological and sensory trade-offs. To ensure that such innovations truly contribute to sustainable diets, future research should focus on refining incorporation techniques, standardizing raw materials, and enhancing consumer engagement, to bridge the gap between nutritional value and palatability.

## 7. Conclusions

The olive oil and wine industries, deeply intertwined in their historical, economic, and environmental contexts, generate substantial organic waste streams, such as olive pomace, grape pomace, and wastewater. These byproducts, which are rich in bioactive compounds such as hydroxytyrosol, resveratrol, and flavonoids, present valuable opportunities for developing functional foods and nutraceuticals within a circular economy framework. Integrating valorization strategies between the two industries can enhance recovery efficiency, foster technological synergies, and support sustainable rural development. However, challenges remain, including the variability in waste composition, technological adaptation, and regulatory alignment. Addressing these issues requires coordinated innovation, governance, and stakeholder engagement to ensure that dual valorization efforts deliver tangible environmental, economic, and public health benefits (Table 5).

## Figures and Tables

**Table 1:** Mass balance of olive components in virgin olive oil extraction process [21,30,31].

Extraction Method	Olive Input (kg)	Oil Output (kg)	OMSW ^1^ (kg)	OMWW ^2^ (kg)
Pressure	1000	190–200	200–400	400–600
Two-Phase Centrifugation	1000	190–210	800–950 (wet pomace)	0–200
Three-Phase Centrifugation	1000	190–210	500–600	1000–1200 (process water included)

^1^ Olive mill solid waste. ^2^ Olive mill wastewater.

**Table 3:** Comparative summary of extraction methods for olive oil industry byproducts.

Extraction Method	Key Conditions	Yields/Advantages
Ultrasound-Assisted Extraction (UAE)	Ultrasonic waves, mild temperature, short time	High yields of phenolic compounds and triterpenes; energy-efficient and eco-friendly
Supercritical Fluid Extraction (SFE)	Supercritical CO_2_ at ~31 °C and 74 bar; sometimes with ethanol as co-solvent	High recovery of fatty acids and triterpenes; solvent-free residue; selective and green
Pressurized Liquid Extraction (PLE)	High temperature (50–200 °C) and pressure (10–15 MPa) with water or ethanol	Efficient for secoiridoids and flavonoids; reduced solvent use and processing time
Microwave-Assisted Extraction (MAE)	Microwave energy, short processing time, solvents (ethanol–water mixtures)	High phenolic content recovery; fast and low energy compared to conventional methods
Enzyme-Assisted Extraction (EAE)	Enzymes (cellulases, hemicellulases, pectinases) at mild temperatures and neutral pH	Enhanced release of bound bioactives; greener approach; improved cell wall degradation
Chromatographic Analysis	HPLC-MS, GC-MS	Analytical purpose: identification and quantification of bioactive compounds

**Table 4:** Extraction methods, polyphenol yields, and main compounds identified in grape and wine industry byproducts.

Byproduct	Extraction Method(s)	Polyphenol Yield (mg GAE/g DW)	Main Polyphenols	Reference
Grape Pomace	Pressurized liquid extraction Microwave-assisted extraction Matrix solid-phase dispersion Pulsed electric field and high-voltage electric discharge	58	Phenolic acids: Benzoic acid; Hydroxycinnamic acid Flavonoids: Catechins; Flavonols; Anthocyanins; Tannins: Proanthocyanidins; Protocatechuic acid; Quercetin-3-O-glucuronide	[92]
Grape Seeds	Pressing Solvent extraction Ultrasound-assisted extraction Supercritical fluid extraction Pressurized liquid extraction	0.11 to 18.48 *	Nonflavonoids: Gallic acid; Hydroxybenzoic acid; p-Coumaric acid; Ferulic acid; Caffeic acid; trans-Cinnamic acid; Resveratrol Flavonoids: Flavan-3-ols: (+)-Catechin and (-)-Epicatechin; Flavonols: Quercetin, Myricetin; Kaempferol Flavanones: Naringenin Flavones: Krisoeriol Isoflavones: Formononetin	[49]
Grape Skins	Water-based extraction at different temperatures Ethanol–water mixture extraction	1.62 to 1.94	Flavanols: Catechin; Epicatechin; Gallocatechin; Epigallocatechin; Epicatechin-gallate Flavonols: Quercetin Anthocyanins: Glucosides; Caffeoyl glucoside; Acetyl glucosides; Coumaroyl glucosides; Pyroanthocyanins (vitisin A and B); Malvidin coumaroyl Phenolic acids: Ethyl gallate; p-Coumaroyl-glucosides; Gallic acid; Galloyl glucose Stilbenoids: Resveratrol glucoside	[38]
Grape Stems	Conventional extraction: solid–liquid and liquid–liquid extractions using solvents Optimized extraction methods: These focus on optimizing parameters like solvent type, time, temperature, and pH to improve extraction yield and efficiency Enzymatic extraction High-temperature extraction	58 **	Flavanols: Catechin; Epicatechin; Procyanidin dimer B1; Procyanidin dimer B3 Flavonols: Quercetin-3-O-glucuronide; Quercetin-3-O-rutinoside Phenolic acids: Gallic acid; Syringic acid; Caftaric acid Anthocyanins: Malvidin-3-O-glucoside Stilbenes: Resveratrol; Σ-viniferin	[90]
Wine Lees	Conventional extraction (maceration): This method typically uses ethanol–water mixtures as solvents Ultrasound-assisted extraction Microwave-assisted extraction Enzyme-assisted extraction Membrane-based extraction Supercritical fluid extraction	3.1 to 58.8	Anthocyanins Flavonoids: Catechin; Epicatechin; Quercetin Phenolic acids: Gallic acid; Resveratrol; Myricetin Pyranoanthocyanins	[79]
Winery Wastewater	Membrane filtration (e.g., ultrafiltration, nanofiltration) Adsorption techniques	-	Phenolic acids: Gallic acid; Syringic acid; Caffeic acid; Chlorogenic acid; Coutaric acid; Fertaric acid; Protocatechuic acid; Trans-caftaric acid; Vanillic acid; Ferulic acid Flavonoids: (-)-Epicatechin; (-)-Epigallocatechin gallate; (+)-Catechin; Quercetin (including quercetin aglycone) Anthocyanins: Cyanidin-3-O-glucoside; Malvidin-3-O-glucoside; Peonidin-3-O-glucoside; Petunidin-3-O-glucoside; Malvidin 3-(acetyl)-glucoside; Malvidin 3-(coumaroyl)-glucoside 12 Resveratrol	[95]

Notes: GAE: gallic acid equivalents; DW: dry weight. Polyphenol yields can vary depending on grape variety, extraction conditions, and analytical methods. * Values were obtained by conversion of 0.93 to 154.0 mg GAE/100 g oil, assuming an average oil yield of 12%. ** Value was obtained knowing that polyphenols can constitute up to 5.8% of the dry mass of grape stems.

**Table 5:** Recommendations for future research and associated challenges.

Aspect	Recommendations for Future Research	Challenges/Limitations
Olive oil byproducts	Investigate the scalability and economic viability of green extraction technologies (e.g., supercritical fluids, ultrasound, microwaves); explore process optimization through metabolomics and improve encapsulation and bioavailability of bioactive compounds.	High investment costs; complex, high-moisture matrices; low yields; regulatory uncertainty; consumer skepticism.
Wine byproducts	Assess cost-effective and scalable extraction methods tailored to grape matrices; conduct market acceptance studies of co-valorized products; explore cooperative business models to integrate value chains.	High energy and infrastructure demands; seasonal and compositional variability; regulatory and market barriers.
Functional food formulation	Develop strategies to standardize raw materials; optimize incorporation levels to balance bioactivity and sensory acceptance; conduct consumer preference and willingness-to-pay studies.	Degradation of bioactives during processing; negative sensory impacts at high inclusion levels; batch variability.
Policy and governance	Promote evidence-based policies supporting economic feasibility and scalability of byproduct valorization; improve transparency and inclusivity in governance frameworks.	Lengthy, costly approval processes for novel foods and health claims; lack of economic models for investment planning.
Socioeconomic integration	Examine the socioeconomic impact of integrated olive oil–wine byproduct valorization; design market strategies that leverage cultural heritage and rural development; explore joint tourism initiatives.	Need for systemic changes in policy, funding mechanisms, and cross-sector coordination.

## Data Availability

No new data were created or analyzed in this study. Data sharing is not applicable to this article.
